# A Robust Supervised Variable Selection for Noisy High-Dimensional Data

**DOI:** 10.1155/2015/320385

**Published:** 2015-06-02

**Authors:** Jan Kalina, Anna Schlenker

**Affiliations:** ^1^Institute of Computer Science of the Czech Academy of Sciences, Pod Vodárenskou Vĕží 2, 182 07 Prague 8, Czech Republic; ^2^Department of Biomedical Informatics, Faculty of Biomedical Engineering, Czech Technical University in Prague, Náměstí Sítná 3105, 272 01 Kladno, Czech Republic

## Abstract

The Minimum Redundancy Maximum Relevance (MRMR) approach to supervised variable selection represents a successful methodology for dimensionality reduction, which is suitable for high-dimensional data observed in two or more different groups. Various available versions of the MRMR approach have been designed to search for variables with the largest relevance for a classification task while controlling for redundancy of the selected set of variables. However, usual relevance and redundancy criteria have the disadvantages of being too sensitive to the presence of outlying measurements and/or being inefficient. We propose a novel approach called Minimum Regularized Redundancy Maximum Robust Relevance (MRRMRR), suitable for noisy high-dimensional data observed in two groups. It combines principles of regularization and robust statistics. Particularly, redundancy is measured by a new regularized version of the coefficient of multiple correlation and relevance is measured by a highly robust correlation coefficient based on the least weighted squares regression with data-adaptive weights. We compare various dimensionality reduction methods on three real data sets. To investigate the influence of noise or outliers on the data, we perform the computations also for data artificially contaminated by severe noise of various forms. The experimental results confirm the robustness of the method with respect to outliers.

## 1. Introduction

Variable selection represents an important category of dimensionality reduction methods frequently used in the analysis of multivariate data within data mining and multivariate statistics. Variable selection with the aim of finding a smaller number of key variables is an inevitable tool in the analysis of high-dimensional data with the number of variables *p* largely exceeding the number of observations *n* (i.e., *n* ≪ *p*) [1, 2]. The requirement to analyze thousands of highly correlated variables measured on tens or hundreds of samples is very common, for example, in molecular genetics. If the observed data come from several different groups and the aim of the data analysis is learning a classification rule, supervised dimensionality reduction methods are preferable [3], because unsupervised methods such as principal component analysis (PCA) cannot take the information about the group membership into account [4].

While real data are typically contaminated by outlying measurements (outliers) caused by various reasons [5], numerous variable selection procedures suffer from the presence of outliers in the data. Robust dimensionality reduction procedures resistant to outliers were proposed typically in the form of modifications of PCA [6–9]. Still, the importance of robust variable selection increases [10] as the amount of digital information worldwide increases unimaginably.

Most of the available variable selection procedures tend to select highly correlated variables [11]. This is also the problem of various Maximum Relevance (MR) approaches [12], which select variables inefficient for classification tasks because of the undesirable redundancy in the selected set of variables [13]. As an improvement, the Minimum Redundancy Maximum Relevance (MRMR) criterion was proposed [14] with various criteria for measuring the relevance of a given variable and redundancy within the set of selected key variables. Its ability to avoid selecting highly correlated variables brings about benefits for a consequent analysis. However, the methods remain too vulnerable to outlying values and noise [15].

In this paper, we propose a new MRMR criterion combining principles of regularization and robust statistics, together with proposing a novel optimization algorithm for its computation. It is called Minimum Regularized Redundancy Maximum Robust Relevance (MRRMRR). For this purpose, we recommend using a highly robust correlation coefficient [16] based on the least weighted squares regression [17] as a new measure of relevance of a given variable. Further, we define a new regularized version of the coefficient of multiple correlation and use it as a redundancy measure. The regularization allows computing it in a numerically stable way for *n* ≪ *p* and is advocated as a denoised method improving robustness properties.

This paper has the following structure.  Section 2 describes existing approaches to the MRMR criterion. Sections 3.1 and 3.2 propose and investigate new methods for measuring redundancy and relevance. The MRRMRR method is proposed in Section 3.3.  Section 4 illustrates the new method on three real high-dimensional data sets. There, we compare various approaches for finding 10 most important genes and compare their ability to discriminate between two groups of samples. The discussion follows in Section 5.

## 2. MRMR Variable Selection

This section critically discusses existing approaches to the MRMR criterion, overviews possible relevance and redundancy measures, and introduces useful notation. The total number *n* of *p*-dimensional continuous data is assumed to be observed in *K* different groups, where *p* is allowed to largely exceed *n*. Let **X** denote the data matrix with *X*_*ij*_ denoting the *j*th variable observed on the *i*th sample, where *i* = 1,…, *n* and *j* = 1,…, *p*. The *j*th variable observed across *n* samples will be denoted by **X**_*j*_ = (*X*_1*j*_,…, *X*_*nj*_)^*T*^ for *j* = 1,…, *p*. Let **Y** = (*Y*_1_,…, *Y*_*n*_)^*T*^ denote the vector of group labels (true group membership), which are values from the set {1,…, *K*}. The aim is to find a small number of variables, which allow solving the classification task into the groups reliably.

In its habitually used form, the MRMR variable selection can be described as a forward search. The set of selected variables will be denoted by *S*, starting with *S* = *∅*. At first, the most relevant single variable is selected to be an element of *S*. Then, such variable is added to *S*, which maximizes a certain criterion combining relevance and redundancy. In such a way, one variable after another is added to *S*. Common criteria for combining relevance and redundancy include their difference or ratio [11, 14, 15] or in a more flexible way

(1) $$\begin{array}{c} Rel\left(\;S\;\right)-\gamma \cdot \mathrm{Red}\left(\;S\;\right) \end{array}$$

with a fixed *γ* ∈ [0,1], while choosing a fixed *γ* ∈ [0.5,1] was recommended by [13].

Relevance of a set of variables *S* is commonly measured as

(2) $$\begin{array}{c} Rel\left(\;S\;\right)=\frac{1}{\left|S\right|}\underset{k\in S}{\sum }\left|\;R_{1}\left(\;Y,X_{k}\;\right)\;\right|, \end{array}$$

where *R*_1_ is a specified measure of similarity (suitable for measuring association between a continuous and a discrete variable), |*S*| is the number of variables in *S*, and the sum is computed over all variables of *S*. Common examples of *R*_1_ include measures based on mutual information [13, 14] or other approaches requiring a discretization (or even dichotomization) of the data [15], the *F* statistic of the analysis of variance [11], or Spearman rank correlation coefficient. Specific ad hoc measures were proposed for *K* = 2 and cannot be easily generalized for *K* > 2.

Redundancy of a set of variables *S* is commonly measured only as a sum of contributions of individual variables

(3) $$\begin{array}{c} Red\left(\;S\;\right)=\frac{1}{{\left|S\right|}^{2}}\underset{k,l\in S}{\sum }\left|\;R_{2}\left(\;X_{k},X_{l}\;\right)\;\right|, \end{array}$$

where *R*_2_ is a specified measure of similarity (suitable for measuring association between two continuous variables). Common examples of *R*_2_ include the mutual information or other measures based on information theory [11, 13, 14], test statistics or *p* values of the Kolmogorov-Smirnov or sign tests, or very simple ad hoc criteria [15]. To the best of our knowledge, no measure able to capture the multivariate structure of the data (e.g., coefficient of multiple correlation) has been used in this context.

Disadvantages or limitations of the MRMR in the habitually used form include a high sensitivity of standard relevance and redundancy measures to the presence of outliers in the data. While nonparametric measures do not suffer from such sensitivity, they remain inefficient for data without contamination by severe noise. Moreover, the mutual information (as well as some other measures) is unsuitable for continuous data. Commonly, continuous data are discretized, which is strongly depreciated due to an unnecessary loss of information [18]. Besides, some authors performed the discretization of continuous data without giving its sufficient description [13], while the effect of discretization of the data has not been systematically examined [15]. In the next section, we propose a robust and efficient version of the MRMR criterion, which uses a suitable regularization and tools of robust statistics.

## 3. Methodology

### 3.1. Regularized Coefficient of Multiple Correlation

Redundancy is a measure of association between a continuous variable **Z** and the whole set *S* of several continuous variables. The coefficient of multiple correlation is suitable to evaluate the linear association between **Z** and the variables in *S* jointly by finding the maximal linear combination of the variables in *S*. In order to allow the method to be feasible also for the number of variables in *S* exceeding *p*, we resort to a regularized coefficient of multiple correlation, which can be also interpreted as a regularized coefficient of determination *R* ^2^ in linear regression of **Z** against all variables included in *S*. While the regularized coefficient may be used as a self-standing correlation measure, it will be used as a redundancy measure within the MRMR criterion in Section 3.3.

Within the computation of the MRMR, the set of selected variables *S* is gradually constructed by including one variable after another, starting with selecting the most relevant single variable, which will be denoted by **T**_1_. In each step, it is necessary to measure the redundancy of *S* after adding a candidate variable **Z** = (*Z*_1_,…, *Z*_*n*_)^*T*^ observed across *n* samples to *S*. After a certain number of *s* steps of the algorithm, there will be exactly *s* variables in *S*. These will be denoted by **T**_1_,…, **T**_*s*_, where the *j*th variable **T**_*j*_ contains data values **T**_*j*_ = (*T*_1*j*_,…, *T*_*nj*_)^*T*^. Let us now consider *s* to be fixed and the aim is to measure association between **Z** and variables **T**_1_,…, **T**_*s*_ jointly. The idea of Tikhonov regularization [19, 20] will be used to obtain a formal definition of a regularized coefficient of multiple correlation.

**Definition 1 .**

Let $\tilde{R}$ denote the empirical correlation matrix computed for the data

(4) $$\begin{array}{c} \left(\begin{array}{cccc} T_{11} & \cdots & T_{1s} & Z_{1}\\ \vdots & \ddots & \vdots & \vdots \\ T_{n1} & \cdots & T_{ns} & Z_{n} \end{array}\right). \end{array}$$

We define its regularized counterpart **R** ^∗^ as

(5) $$\begin{array}{c} R^{*}=\left(\;1-\lambda \;\right)\tilde{R}+\lambda I_{s+1},\;\lambda \in \left(0,1\right), \end{array}$$

where *I*_*s*+1_ denotes a unit matrix of size (*s* + 1)×(*s* + 1).

The matrix **R** ^∗^ is ensured to be regular even for *n* ≪ *p*. In the whole work, we will work only with the asymptotically optimal value of *λ*, which minimizes the mean square error of **R** ^∗^ over *λ* ∈ (0,1). This will be denoted by *λ* ^∗^ and is obtained by modifying the general result of [21] to our context. For the sake of simplifying the notation, let **T**_*s*+1_ denote the candidate variable **Z**. Then, assuming *s* → *∞*, the explicit expression for *λ* ^∗^ is distribution-free and is equal to

(6) $$\begin{array}{c} \lambda^{*}=\frac{2\sum_{i=2}^{s+1}\;\sum_{j=1}^{i-1}\hat{\mathrm{var}}\left(S_{ij}^{*}\right)}{2\sum_{i=2}^{s+1}\;\sum_{j=1}^{i-1}{\left(S_{ij}^{*}\right)}^{2}}, \end{array}$$

where

(7) Sij∗=cov⁡Ti,Tj,i=1,…,s+1,  j=1,…,s+1,var^⁡Sij∗=nn−13∑k=1s+1Tki−T−i2Tkj−T−j2−s+1n2∑k=1s+1Tki−T−iTkj−T−j2,T−i=1n∑j=1nXki,i=1,…,s,T−s+1=1n∑k=1nZk.

Let us denote elements of **R** ^∗^ computed with *λ* ^∗^ by

(8) $$\begin{array}{c} \left(\begin{array}{cccc} r^{*}\left(T_{1},T_{1}\right) & \cdots & r^{*}\left(T_{1},T_{s}\right) & r^{*}\left(T_{1},Z\right)\\ \vdots & \ddots & \vdots & \vdots \\ r^{*}\left(T_{s},T_{1}\right) & \cdots & r^{*}\left(T_{s},T_{s}\right) & r^{*}\left(T_{s},Z\right)\\ r^{*}\left(Z,T_{1}\right) & \cdots & r^{*}\left(Z,T_{s}\right) & r^{*}\left(Z,Z\right) \end{array}\right), \end{array}$$

where diagonal elements are equal to 1. We will use the components of (8) to define **R**_**TZ**_^∗^ and **R**_**TT**_^∗^ by

(9) RZT∗r∗T1,Z,…,r∗Ts,ZT,RTT∗=r∗T1,T1⋯r∗T1,Ts⋮⋱⋮r∗Ts,T1⋯r∗Ts,Ts.

**Definition 2 .**

Let the regularized coefficient of multiple correlation between the vector **Z** and the set of vectors {**T**_1_,…, **T**_*s*_} be defined as

(10) $$\begin{array}{c} {\tilde{r}}^{*}\left(\;Z,T\;\right)=\sqrt{{\left(R_{ZT}^{*}\right)}^{T}{\left(R_{TT}^{*}\right)}^{-1}R_{ZT}^{*}}. \end{array}$$

We stress that (9) can be computed only after computing the whole matrix **R** ^∗^. For example, *r* ^∗^(**T**_1_, **T**_2_) depends also on **T**_3_,…, **T**_*s*_ and **Z**. In other words, variables with a large variability borrow information from more stable (less variable) variables in a way analogous to [22] and ${\tilde{r}}^{*}$ can be considered to be a denoised version of its classical counterpart. Besides, (5) can be interpreted also from other points of view:

(i) It can be motivated as an attempt to correct for an excessive dispersion of sample eigenvalues of the empirical correlation matrix of **T**_1_,…, **T**_*s*_, similarly to [23].
(ii) Equation (5) is a regularized estimator of the correlation matrix shrunken towards a unit matrix. This biased estimator with the optimal value of *λ* has a smaller quadratic risk compared to its classical counterpart thanks to Stein's paradox [24, 25]. This explains why a regularized estimator brings about benefits also if the set *S* is chosen to be relatively small (e.g., 10 variables).
(iii) From the point of view of robust optimization [26], (5) can be interpreted as locally robust against small departures in the observed data.
(iv) Equation (5) can be derived as a Bayesian estimator, assuming the inverse of the population counterpart of **S** ^∗^ to follow a Wishart distribution with a diagonal expectation (cf. [21]).

**Remark 3 .**

The matrix **R**_**TT**_^∗^ is always regular. Denoting eigenvalues of the empirical correlation matrix computed from data

(11) $$\begin{array}{c} \left(\begin{array}{ccc} T_{11} & \cdots & T_{1s}\\ \vdots & \ddots & \vdots \\ T_{n1} & \cdots & T_{ns} \end{array}\right) \end{array}$$

by *θ*_1_,…, *θ*_*s*_, the fact follows from the explicit formula for the eigenvalues of **R**_**TT**_^∗^ in the form (1 − *λ* ^∗^)*θ*_*i*_ + *λ* ^∗^ for *i* = 1,…, *p*; that is, they are positive.

**Remark 4 .**

An efficient computation of (10) can exploit the singular value decomposition of **R**_**TT**_^∗^ in the form **R**_**TT**_^∗^ = **Q**Θ**Q** ^*T*^, where Θ is diagonal and **Q** is an orthogonal matrix. Particularly,

(12) $$\begin{array}{c} {\left(\;R_{TT}^{*}\;\right)}^{-1}=Q\Theta^{-1}Q^{T}, \end{array}$$

where

(13) Θ−1=diag⁡1−λ∗θ1+λ∗−1,…,1−λ∗θs+λ∗−1.

### 3.2. Robust Correlation Coefficient

In this section, some properties of the robust correlation coefficient *r*_LWS_ [16] based on the least weighted squares (LWS) regression are derived and we recommend using *r*_LWS_ as a relevance measure for the MRMR criterion for samples coming from *K* = 2 groups.

The LWS estimator [17] is a robust estimator of regression parameters in linear regression model with a high finite-sample breakdown point [5, 27], that is, highly robust against severe outliers in the data. If the quantile-based adaptive (data-dependent) weights of [28] are used, the estimator attains a full asymptotic efficiency of the least squares (i.e., for noncontaminated normal data). The LWS estimator can be computed using a weighted version of the fast algorithm of [29].

Based on the LWS estimator for the linear regression, a robust correlation coefficient *r*_LWS_(**U**, **V**) was proposed by [16] as a measure of linear association between two data vectors

(14) UU1,…,UnT,V=V1,…,VnT,

in the linear regression model

(15) $$\begin{array}{c} V_{i}=\beta_{0}+\beta_{1}U_{i}+e_{i},\;i=1,\ldots ,n. \end{array}$$

Assuming data (14) to follow a continuous distribution, the appealing properties of *r*_LWS_ are inherited from the LWS estimator [16]. To avoid confusion, let us introduce a special notation for various versions of the robust correlation coefficient *r*_LWS_ based on different choices of weights.

**Definition 5 .**

One uses the notation *r*_LWS_^*A*^(**U**, **V**) to define *r*_LWS_(**U**, **V**) with the adaptive weights of [28]. The notation *r*_LWS_^LD^(**U**, **V**) is used for *r*_LWS_(**U**, **V**) computed with the linearly decreasing weights and the notation *r*_LWS_^Log^(**U**, **V**) is used for *r*_LWS_(**U**, **V**) computed with weights defined by means of a logistic decreasing function [16].

The value of *r*_LWS_ is a measure of goodness of the linear fit in (15). We will now derive some properties of *r*_LWS_^*A*^, which are inherited from properties of the LWS regression estimator. The computation of *r*_LWS_^*A*^ requires computing an initial highly robust estimator of **β** = (*β*_0_, *β*_1_)^*T*^ in (15); this can be, for example, the least trimmed squares (LTS) estimator [30].

**Theorem 6 .**

Let (*U*_1_, *V*_1_)^*T*^,…, (*U*_*n*_, *V*_*n*_)^*T*^ be a sequence of independent identically distributed random vectors with *n* > 2. One assumes any two observations to give a unique determination of **β** in the linear regression of **V** against **U** almost surely. Let *ϵ*_*n*_^0^ denote the finite-sample breakdown point of an initial estimator of **β** in (15). Then the finite-sample breakdown point of *r*_*LWS*_^*A*^ is larger than or equal to

(16) $$\begin{array}{c} \left\{\;\epsilon_{n}^{0},\frac{\left\{\left\lfloor \left(n+1\right)/2\right\rfloor -2\right\}}{n}\;\right\}. \end{array}$$

**Proof**

The finite-sample breakdown point of *r*_LWS_^*A*^ corresponds to the smallest percentage of data that may be arbitrarily contaminated causing *r*_LWS_^*A*^ to take an arbitrary large aberrant value (to “break down”) [31]. The robust correlation coefficient inherits the breakdown point of the LWS estimator, which was derived by [28] for the linear regression with *p* regressors to be

(17) $$\begin{array}{c} \left\{\;\epsilon_{n}^{0},\frac{\left\{\left\lfloor \left(n+1\right)/2\right\rfloor -\left(p+1\right)\right\}}{n}\;\right\}. \end{array}$$

Now we study the asymptotic distribution of the robust correlation coefficient based on the LWS estimator under technical (but very general) assumptions.

**Theorem 7 .**

One considers the data (*U*_1_, *V*_1_)^*T*^,…, (*U*_*n*_, *V*_*n*_)^*T*^ as a random sample from a bivariate normal distribution with correlation coefficient *ρ*. One assumes the assumptions of Theorem 3 of [28] to be fulfilled. Then, for *n* → *∞*, *r*_*LWS*_ converges in distribution to a random variable following normal distribution. Specifically, the asymptotic distribution of *r*_*LWS*_^*A*^ can be approximated by

(18) $$\begin{array}{c} N\left(\;\rho ,\frac{{\left(1-\rho \right)}^{2}}{n}\;\right) \end{array}$$

under the assumption *ρ* ∈ (−1,1).

**Proof**

The convergence to the normal distribution for *n* → *∞* follows from the asymptotic normality of **b**_LWS_ with adaptive weights [28] and from the expression

(19) $$\begin{array}{c} r_{\text{LWS}}^{A}=b_{1}^{\text{LWS}}\sqrt{\frac{\sum_{i=1}^{n}{\tilde{w}}_{i}{\left(U_{i}-{\overset{-}{U}}_{\text{LWS}}\right)}^{2}}{\sum_{i=1}^{n}{\tilde{w}}_{i}{\left(V_{i}-{\overset{-}{V}}_{\text{LWS}}\right)}^{2}}}, \end{array}$$

where ${\tilde{w}}_{1},\ldots ,{\tilde{w}}_{n}$ are weights determined by the LWS regression in (15) and ${\overset{-}{U}}_{\text{LWS}}$ and ${\overset{-}{V}}_{\text{LWS}}$ are weighted means computed with these weights. The asymptotic expectation and variance of *r*_LWS_ are equal to the expectation and variance of the sample correlation coefficient, which were approximated by [32].

Pearson's correlation coefficient *r*(**U**, **V**) is a valid relevance measure also if **V** is binary. Indeed, robust correlation measures have been used in the context of logistic regression [33]. This makes *r*_LWS_ suitable also within the MRMR criterion for measuring association between a binary vector of labels (group membership) and a continuous data vector for *K* = 2. In this context, *r*_LWS_ ensures a high robustness with respect to outliers in the continuous variable **X**_*k*_ in (2), where the vector of labels is considered to be its response.

### 3.3. MRRMRR Variable Selection

We introduce a new version of the MRMR criterion using a regularized redundancy measure of Section 3.1 and a robust relevance measure of Section 3.2. It is denoted as Minimum Regularized Redundancy Maximum Robust Relevance (MRRMRR) and can be interpreted as insensitive to the presence of outliers in the continuous measurements **X**.

We search for the optimal value of *γ* in (1), which allows the best classification performance over all possible *γ* > 0. Because the relevance and redundancy may not be directly comparable or standardized to the same limits, we do not require *γ* ≤ 1.

**Algorithm 8 .**

Put *S* = *∅*. First, the most relevant variable is selected using (2) and is included in the set of variables *S*. Further, the following procedure is repeated. Let **X**_*k*_ denote the expressions of the *k*th variable in *S* across observations. We add such variable **Z** = (*Z*_1_,…, *Z*_*n*_)^*T*^ not included in *S* to the set *S*, which maximizes the criterion

(20) $$\begin{array}{c} \max\left[\;\left|\;r_{\text{LWS}}\left(Y,Z\right)\;\right|-\gamma \underset{k\in S}{\sum }\left|\;{\tilde{r}}^{*}\left(X_{k},Z\right)\;\right|\;\right], \end{array}$$

over all variables not included in *S* and over all values of *γ* ≥ 0. Other variables are included step by step to *S*, until *S* contains a fixed number of variables, determined before the computations. This approach is repeatedly applied with different fixed values of *γ* and such value of *γ* is found optimal, which allows the best classification performance.

Concerning the optimal number of selected variables, we refer to [11] for a discussion. Basically, a fixed number of the top-ranked genes are commonly selected to yield the classification error equal to a specified constant [14]. Other works applied an intuitive trial and error approach for specifying a fixed number of selected variables without supporting the choice by rigorous arguments.

## 4. Results

We compare the performances of various MRMR criteria on three real data sets.

### 4.1. Cardiovascular Genetic Study

We use gene expression data set from a whole-genome study on 24 patients immediately after a cerebrovascular stroke (CVS) and 24 control persons. This study of the Center of Biomedical Informatics in Prague (2006–2011) had the aim of finding a small set of genes suitable for diagnostics and prognosis of cardiovascular diseases. The data for *p* = 38 614 gene transcripts were measured using HumanWG-6 Illumina BeadChip microarrays. The study complies with the Declaration of Helsinki and was approved by the local ethics committee.

We perform all computations in R software. Variable selection (gene selection) is performed by means of various MRMR criteria with a fixed *γ* with the requirement to find 10 most important genes. We use the following relevance measures: mutual information, Pearson correlation coefficient *r*, Spearman rank correlation coefficient *r*_*S*_, and robust correlation coefficients *r*_LWS_^*A*^, *r*_LWS_^LD^, and *r*_LWS_^Log^ (Definition 5). Redundancy is evaluated using (3), where *R*_2_ has the form of mutual information, *r*, *r*_*S*_, *p* value of the Kolmogorov-Smirnov test, *p* value of the sign test, and ${\tilde{r}}^{*}$.

Classification performance on a reduced set of variables obtained by various dimensionality reduction procedures is evaluated by means of a leave-one-out cross validation. For this purpose, the data are repeatedly divided into training (47 individuals) and validation sets (1 individual). The classification rule of the linear discriminant analysis (LDA) is learned over the training set and is applied to classify the validation set. This is repeated 48 times over all possible choices of the training set, computing the values of sensitivity and specificity of the classification procedures for each case. At the same time, we compute the classification accuracy with the optimal *γ* ≥ 0. Classification accuracy is equal to half of the sum of sensitivity and specificity, that is, the number of correctly classified cases divided by the total number of cases, obtained with the optimal *γ* (over *γ* ≥ 0).

Various other classification methods are used without a prior dimensionality reduction, including Prediction Analysis for Microarrays (PAM) [22], shrunken centroid regularized discriminant analysis (SCRDA) [19], and support vector machines (SVM). For comparison, we investigate also the effect of dimensionality reduction by means of PCA.

Table 1 presents results for some fixed values of *γ* as well as results obtained with the optimal value of *γ* according to Algorithm 8, that is, that nonnegative *γ* maximizing the classification accuracy over all its possible values. In all versions of the MRMR approach, the optimal classification was obtained with *γ* ≤ 0.9. The results in Table 1 reveal that MRRMRR outperforms other approaches to MRMR variable selection. The mutual information turns out to perform even much worse than the correlation coefficient, which is a consequence of discretizing continuous data. Besides, we performed also additional computations, including a 12-fold cross validation, which yields analogous results.

Further we investigate whether the new MRRMRR method can be accompanied by a consequent classification by tools other than LDA. The results are overviewed in Table 2. Clearly, MRRMRR does not seem to be linked to any specific classification tool. SVM as well as SCRDA seem to perform very reliably if accompanied by MRRMRR. An attempt for explanation will follow in Section 5.

In addition, we perform a sensitivity study comparing various versions of the MRMR criterion on the same data artificially contaminated by noise, which was generated as a random variable independently of variable and observation and added to each of the observed data values. For each of the following three distributional models, the noise was generated 100 times:

(i) Noise 1: normal distribution *N*(0,0.1).
(ii) Noise 2: contaminated normal distribution with cumulative distribution function (c.d.f.) Δ*F* + (1 − Δ)*G*, where Δ = 0.85, *F* is a c.d.f. of *N*(0,0.01), and *G* is a c.d.f. of *N*(0,1).
(iii) Noise 3: Cauchy distribution with probability density function
  (21) $$\begin{array}{c} f\left(\;x\;\right)=\frac{c}{\pi \left(x^{2}+c^{2}\right)},\;x\in R,\;\;c=0.002. \end{array}$$

We used again various MRMR criteria to find the 10 most relevant genes. The classification accuracy of LDA and other methods is compared in a leave-one-out cross validation study.

Averaged results obtained with the optimal *γ* (requiring *γ* ≥ 0) are given in Table 3. They reveal a high vulnerability of available dimensionality reduction methods to the presence of noise. Here, MRRMRR outperforms MRMR with various classical relevance and redundancy measures. Besides, MRRMRR followed by LDA performs comparably to some other standard classification methods, although it actually uses 10 genes, while the other methods (SCRDA, lasso-LR, and SVM) are allowed to use all *p* = 38 614 genes. This performance is verified for noise under all three distributional assumptions and the selected 10 genes by the MRRMRR method do not suffer from noise. The difference between different weight selections for the robust correlation coefficient seems to play only a marginal role and we can say that *r*_LWS_^*A*^ is able to slightly outperform *r*_LWS_^LD^ and *r*_LWS_^Log^.

### 4.2. Metabolomic Profiles Study

We analyze the prostate cancer metabolomic data set of [34], which contains *p* = 518 metabolites measured over two groups of patients, namely, those with a benign prostate cancer (16 patients) and with other cancer types (26 patients). The task in both examples is to learn a classification rule allowing discrimination between *K* = 2 classes of individuals.

Standard classification methods are used on raw data as well as after performing a dimensionality reduction. We use MRRMRR with |*r*_LWS_^*A*^| as the relevance measure and $\left|{\tilde{r}}^{*}\right|$ as the redundancy measure, because such choice turned out to provide the most reliable results for contaminated data in the study on contaminated data in Section 4.1. Results of classification performance in a leave-one-out cross validation study are given in Table 2.

Standard classification methods are able to perform reliably in this data set [35] but do not allow a clear interpretation. Classification performed on the 20 main principal components loses its power, due to the unsupervised nature of PCA. MRRMRR with 20 selected variables allows performing a reliable classification, without losing important information for the classification task.

### 4.3. Keystroke Dynamics Study

Finally, we analyze our keystroke dynamics data of [36] from a study aiming at person authentication based on writing medical reports within a hospital. We proposed and implemented a software system based on keystroke dynamics measurements [37], inspired by biometric authentication systems for medical reports [38, 39].

The training data contain keystroke durations and keystroke latencies measured in milliseconds on 32 probands, who typed a short password (“kladruby”) 10 times in their habitual speed. In spite of a small value of *p* = 15 variables, the data are high-dimensional because *p* exceeds the number of measurements for each individual and we must expect that learning the classification rule would suffer from the curse of dimensionality. In the practical application, one of the 32 individuals identifies himself/herself (say as *XY*) and types the password. The aim of the analysis is to verify whether the individual typing on the keyboard is or is not the person *XY*. Thus, the authentication task is a classification problem to assign the individual to one of the *K* = 2 groups.

Results of classification performance in a leave-one-out cross validation study are given in the last column of Table 2. If the classification is performed with raw data, an SVM outperforms other methods. However, its disadvantages include the inability to find optimal values of their parameters as well as a large number of support vectors [1]. If MRRMRR is used to select 4 variables with |*r*_LWS_^*A*^| as the relevance measure and $\left|{\tilde{r}}^{*}\right|$ as the redundancy measure, there seems to be no major loss of important information for the classification task.

## 5. Discussion

Variable selection represents an irreplaceable tool in the analysis of high-dimensional data, preventing numerous approaches of multivariate statistics and data mining from overfitting the data or even from being computationally infeasible due to the curse of dimensionality. Various versions of the Minimum Redundancy Maximum Relevance approach have been described in references as a supervised variable selection methodology tailor-made for classification purposes, while its primary disadvantage has been explained as its high sensitivity to the presence of outlying measurements [15].

This paper proposes a new version of the MRMR criterion in the form (20) capturing the multivariate structure of the data. The new criterion denoted as the Minimum Regularized Redundancy Maximum Robust Relevance (MRRMRR) is constructed from two essential tools and the robustness of the criterion is given by robustness of both tools. One of them is a relevance measure in the form of a robust correlation coefficient *r*_LWS_^*A*^, for which we investigate theoretical properties. The other is a redundancy measure in the form of a new regularized version of the coefficient of multiple correlation ${\tilde{r}}^{*}$, which can be interpreted as a regularized coefficient of determination in linear regression. They are robust to the presence of noise in the data, numerically stable, and also statistically robust in terms of the breakdown point, that is, to the presence of outliers. Our work is a first attempt to investigate robust and regularized methods within the MRMR criterion, which is limited only to two groups of samples.

Section 4 of this paper illustrates the performance of MRRMRR on three real high-dimensional data sets with different values of *p*. Because the forward search of the MRMR criterion with various choices of relevance and redundancy depends on parameter *γ* in (1), the optimal result is obtained by maximizing the classification accuracy over different values of *γ*. MRRMRR yields very reliable results on the observed data, while there seems to be a negligible difference among the three choices of weights for the implicitly weighted relevance measure (|*r*_LWS_^LD^|, |*r*_LWS_^Log^|, and |*r*_LWS_^*A*^|).

To show the robustness of MRRMRR, the data of Section 4.1 are contaminated again after being contaminated by severe noise. MRRMRR performs as the most robust approach among other variable selection procedures, while the choice of the weights for the robust relevance measure seems to play a negligible role. On the other hand, the vulnerability of some approaches (e.g., mutual information within the MRMR variable selection) has not been sufficiently discussed in references.

In the numerical examples, we also inspected the question: Which classification methods are the most recommendable to accompany the MRRMRR variable selection? Based on the results, SVM, LDA, and SCRDA seem to be suitable for this context, because they allow taking the covariance structure of the data into account. They are reliable also for highly correlated variables, while a prior using of MRRMRR avoids their specific disadvantages characteristic for high-dimensional data. On the other hand, MRRMRR does not bring about benefit to classification methods which are based on one-dimensional principles. These include classification trees, PAM (i.e., diagonalized LDA), and others not used in our computations (e.g., Naïve Bayes classifier).

The regularization used in (5) is a popular tool allowing modifying statistical methods for the context of high-dimensional data. As Section 4.3 reveals, regularization brings about benefits for multivariate data also with a small number of variables. Thus, the regularization of Section 3.1 turns out to be suitable also for high-dimensional data with any *p*. Also in a general setting, regularization has been described as a finite-sample (nonasymptotic) approach for multivariate data, not limited to the context of high-dimensional data [1, 24].

Every version of the MRMR method allows finding a set containing a fixed number of genes, which must be chosen before the computation. In the examples, we used an arbitrary choice mainly for comparison purposes. In practice, a more flexible approach would be to use the optimal number of variables according to a criterion evaluating the contribution of the variables to the classification problem taking the total number of variables into account [15].

Other possible relevance measures not studied in the references include measures based on nonparametric analysis of variance (e.g., Kruskal-Wallis, van der Waerden, and median tests [40]), logistic regression (probability of belonging to group 1 or deviance), or a coefficient of determination corresponding to ridge regression or lasso estimators [1]. A natural extension of our approach to several (*K* ≥ 2) groups would be to replace the robust correlation coefficient with a highly robust version of the analysis of variance.

As a limitation of the MRRMRR approach compared to other MRMR approaches, its higher computational complexity compared to simple approaches of (1) with a fixed *γ* must be mentioned. Besides, the idea of Tikhonov regularization (5) is tailor-made for data with variables of the same type, for example, variables measures in the same units and with a similar level of variability. This may not be adequate if the observed variables are very heterogeneous. Other limitations of MRRMRR include those common to all MRMR approaches. Particularly, it does not possess a high stability like other variable selection procedures [41] and a too small number of selected variables in the MRRMRR approach may be criticized for its limited classification ability [18, 42].

The MRRMRR method is primarily designed as a variable selection tool, tailor-made for data which are observed in two different groups. Thus, if the very aim of the high-dimensional data analysis is classification analysis without an explicit need for a variable selection, the user may prefer to use classification methods directly, that is, those which are reliable for *n* ≪ *p*. These direct classification methods not requiring a prior dimensionality reduction (regularized LDA of [19] or SVM) may yield comparable (or possibly even better) results, but we stress their different primary aim. On the other hand, if the very aim of the analysis is comprehensibility of the classification approach, the user may want to avoid the classifiers in the form of a black box. In such situations, the new MRRMRR variable selection represents a suitable tool, which is robust to the presence of outlying values.

## Figures and Tables

**Table 1:** Leave-one-out cross validation performance of various classification methods for the data of Section 4.1. MRMR is used in version (1) or (20) to find 10 variables, while the optimal *γ* over all *γ* ≥ 0 is used. Sensitivity (SE) and specificity (SP) are given for selected fixed values of *γ*.

Dimensionality reduction			Classif. method	Classif. accuracy								

MRMR variable selection												
Measure of		MRMR criterion				Parameter *γ*						
relev.	redund.					0	0.1	0.2	0.3	0.5	0.7	0.9

Mutual info.	Mutual info.	(1)	LDA	0.92	SE	0.75	0.83	0.92	0.88	0.96	0.96	0.96
					SP	0.67	0.92	0.88	0.92	0.96	0.92	0.92

|*r*|	| *r* |	(1)	LDA	1.00	SE	0.92	0.92	0.83	0.88	0.96	0.96	0.96
					SP	0.88	0.96	0.96	0.96	0.96	1.00	1.00

|*r*_*S*_|	|*r*_*S*_|	(1)	LDA	0.96	SE	0.83	0.83	0.96	0.83	0.92	0.96	0.96
					SP	0.88	0.88	0.83	0.96	1.00	0.96	1.00

|*r*|	K-S	(1)	LDA	0.82	SE	0.92	0.92	0.92	0.92	0.92	0.88	0.88
					SP	0.88	0.88	0.88	0.88	0.88	0.96	0.96

|*r*|	Sign test	(1)	LDA	0.82	SE	0.92	0.92	0.92	0.92	0.92	0.88	0.88
					SP	0.88	0.88	0.88	0.88	0.88	0.96	0.96

|*r*|	$\left|{\tilde{r}}^{*}\right|$	(20)	LDA	1.00	SE	0.92	0.92	0.88	0.88	0.92	0.96	1.00
					SP	0.88	0.96	0.96	0.96	0.96	0.96	1.00

|*r*_LWS_^LD^|	$\left|{\tilde{r}}^{*}\right|$	(20)	LDA	1.00	SE	0.92	0.92	0.96	0.96	0.96	0.96	1.00
					SP	0.88	0.88	0.88	0.88	0.92	0.96	1.00

|*r*_LWS_^log⁡^|	$\left|{\tilde{r}}^{*}\right|$	(20)	LDA	1.00	SE	0.92	0.92	0.96	0.96	0.96	0.96	1.00
					SP	0.88	0.88	0.92	0.92	0.92	0.96	1.00

|*r*_LWS_^*A*^|	$\left|{\tilde{r}}^{*}\right|$	(20)	LDA	1.00	SE	0.92	0.92	0.96	0.96	0.96	0.96	1.00
					SP	0.88	0.88	0.92	0.92	0.96	0.96	1.00

**Table 2:** Leave-one-out cross validation performance evaluated by classification accuracy for the data of Sections 4.1, 4.2, and 4.3. MRRMRR uses |*r*_LWS_^*A*^| as the relevance measure and $\left|{\tilde{r}}^{*}\right|$ as the redundancy measure.

Dimensionality reduction	Classification method	Classification accuracy		
		Section 4.1	Section 4.2	Section 4.3
—	SVM	1.00	1.00	0.93
—	Classification tree	0.94	0.97	0.55
—	LDA	Infeasible	Infeasible	Infeasible
—	PAM	0.85	0.98	0.75
—	SCRDA	1.00	1.00	0.79

Number of principal components		10	20	4

PCA	SVM	0.75	1.00	0.90
PCA	Clas. tree	0.72	0.97	0.59
PCA	LDA	0.57	0.90	0.79
PCA	PAM	0.64	0.81	0.77
PCA	SCRDA	0.71	0.92	0.79

Number of variables for MRRMRR		10	20	4

MRRMRR	SVM	1.00	1.00	0.93
MRRMRR	Clas. tree	0.76	0.97	0.55
MRRMRR	LDA	0.95	1.00	0.79
MRRMRR	PAM	0.82	0.97	0.75
MRRMRR	SCRDA	1.00	1.00	0.79

**Table 3:** Leave-one-out cross validation performance evaluated by classification accuracy for the data of Section 4.1 contaminated by noise of three different types. MRMR is used in version (1) or (20) in the same way as in Table 1 to find 10 variables, while the optimal *γ* over all *γ* ≥ 0 is used.

Dimensionality reduction		Classif. method	Noise 1 (normal)	Noise 2 (contam. normal)	Noise 3 (Cauchy)
MRMR variable selection					
Measure of			Classification accuracy		
relev.	redund.				

Mutual info.	Mutual info.	LDA	0.79	0.88	0.92
|*r*|	|*r*|	LDA	0.92	0.85	0.96
|*r*_*S*_|	|*r*_*S*_|	LDA	0.92	0.92	0.96
|*r*|	K-S	LDA	0.92	0.83	0.89
|*r*|	Sign test	LDA	0.84	0.91	0.87
|*r*|	$\left|{\tilde{r}}^{*}\right|$	LDA	0.90	0.86	0.94
|*r*_LWS_^LD^|	$\left|{\tilde{r}}^{*}\right|$	LDA	1.00	1.00	0.98
|*r*_LWS_^log⁡^|	$\left|{\tilde{r}}^{*}\right|$	LDA	1.00	1.00	0.98
|*r*_LWS_^*A*^|	$\left|{\tilde{r}}^{*}\right|$	LDA	1.00	1.00	1.00

Unsupervised dimensionality reduction					
PCA (with 10 princ. components)		LDA	0.79	0.74	0.78

No dimensionality reduction					
—		LDA	Infeasible	Infeasible	Infeasible
—		PAM	0.79	0.73	0.79
—		SCRDA	1.00	1.00	1.00
—		lasso-LR	1.00	1.00	1.00
—		SVM	1.00	1.00	1.00
